# The associations between thyroid-related hormones and the risk of thyroid cancer: An overall and dose-response meta-analysis

**DOI:** 10.3389/fendo.2022.992566

**Published:** 2022-12-07

**Authors:** Zheng Wang, Yuxin Lin, Yixian Jiang, Rong Fu, Yabing Wang, Qian Zhang

**Affiliations:** ^1^ Department of Thyroid and Breast Surgery, Yijishan Hospital of Wannan Medical College, Wuhu, Anhui, China; ^2^ Department of Epidemiology and Health Statistics, School of Public Health, Fujian Medical University, Fuzhou, Fujian, China

**Keywords:** thyroid-related hormones, thyroid-stimulating hormone, free triiodothyronine, free thyroxine, thyroid cancer, meta analysis, dose-response

## Abstract

**Objective:**

Thyroid cancer (TC) is one of the most common malignant tumours of the endocrine system. Thyroid-stimulating hormone (TSH) is known as being a risk factor for TC, but other thyroid-related hormones are inconsistently associated with TC. The purpose of this study was to comprehensively evaluate the relationships between thyroid-related hormones and the risk of TC.

**Methods:**

This study utilized searches of PubMed, Embase, Web of Science and Cochrane library up to the date of March 31st, 2022. Additionally, we performed a systematic review of related original studies combining overall and dose–response meta-analyses.

**Results:**

A total of 30, 5 and 7 articles were included in the meta-analyses of TSH, Free triiodothyronine (FT3), free thyroxine (FT4) and TC risk with 58437, 6813 and 7118 participants respectively. An increased risk of TC was associated with high TSH exposure (OR=1.28, 95% CI: 1.19-1.37, P < 0.001) in the overall meta-analysis. For every 1 mU/L increase in TSH, the risk of TC increased by 16%. However, in those studies that used healthy subjects as controls, the association was not statistically significant(P=0.62). Additionally, high serum FT3 demonstrated a reduced risk of TC, with a combined OR of 0.86 in the fixed-effect model (95% CI: 0.81–0.90, P < 0.001). In addition, a statistically significant increase in TC risk was found when FT4 concentrations reached a certain threshold (approximately 2.2 ng/dL) in the dose-response meta-analysis.

**Conclusions:**

Significant associations between thyroid-related hormones and the risk of TC were found in this study. Further research is needed to understand the underlying mechanisms.

## Introduction

Thyroid cancer (TC) is one of the most common malignant cancers of the endocrine system. The most common pathological types of TC include papillary thyroid carcinoma (PTC) and follicular thyroid carcinoma (FTC), which are differentiated thyroid carcinomas (DTCs). In recent years, the rapid increase in the incidence of TC has made it an important public health problem (1). In the United States, the Surveillance, Epidemiology, and End Results-9 Cancer Registry (SEER-9) data showed that the age-adjusted incidence of TC had rapidly increased from 4.56 cases per 100,000 person-years to 14.42 cases per 100,000 person-years from 1974 to 2013 (2). These increases are mainly due to advances in screening techniques, which have demonstrated increased incidences of small and indolent PTCs (3). However, a previous study reported that incidence-based mortality has also increased in TC, especially in advanced PTC, which is the most common subtype that accounts for 80% of all thyroid carcinomas (2). Therefore, the exploration of risk factors, early diagnoses, and early treatments are of great importance to the prevention and treatment of TC.

The aetiology of TC currently remains unclear. A large number of studies have reported of related factors of TC, such as age, female sex, radiation exposure, and BRAF V600E mutation, which are more or less related to the pathogenesis and clinical features of TC (4–6). In recent years, an increasing number of studies have also reported the association of thyroid-related hormones with TC, but no clear conclusions have been made thus far (7, 8). Thyroid-stimulating hormone (TSH) is the most studied thyroid-related hormone and is the major growth factor for thyroid cells. Moreover, it may act as a cancer stimulus in combination with oncogenes and other growth factors in the growth and development of TC (9, 10). Previous meta-analyses (11, 12) conducted several years ago have explored the association of TSH with TC risk and found that the risk of DTCs significantly increases in parallel with TSH concentrations. In recent years, there has been an increase in original research studies that needs to be updated. The most recent meta-analysis (13) only included studies in which TSH was a continuous variable, and the results of the analysis lacked information on studies with multicategory variables.

Current thyroidology clinical studies that measured thyroid status mainly refer to TSH levels rather than thyroid hormones. In the normal hypothalamic-pituitary-thyroid axis, TSH controls the production and secretion of triiodothyronine and thyroxine and inversely regulates TSH release. After hydrolysis, more than 99% of triiodothyronine and thyroxine form noncovalent bonds with plasma proteins in the circulating blood, thus leaving only a small fraction of free triiodothyronine (FT3) and free thyroxine (FT4), which enter the target cell and bind to the receptor to exert effects (14). A study (15) conducted in 2020 assessed the associations of thyroid hormone levels and TSH levels with a wide range of clinical parameters (such as atrial fibrillation cancer and mortality). The results indicated that thyroid hormone levels appeared to correlate more strongly with clinical parameters than TSH levels. Therefore, TSH and thyroid hormones (such as FT3 and FT4) should be used in combination to assess thyroid function.

Given the high incidence of TC, a comprehensive systematic review and meta-analysis of the associations between thyroid-related hormone levels and the risk of TC is warranted, especially for thyroid hormones (FT3 and FT4), which have not been reported thus far in the literature. Therefore, we synthesized data from the original study and performed overall and dose–response meta-analyses to investigate the association between thyroid-related hormone (TSH, FT3, and FT4) concentrations and the risk of TC.

## Materials and methods

### Publication search

We searched the PubMed, Embase, Web of Science and Cochrane library for all articles on the association between thyroid-related hormones and the risk of TC. The following keywords were used: “thyroid-stimulating hormone”, “thyrotropin”, “thyroid-stimulating hormone”, “free triiodothyronine”, “free thyroxine”, “TSH”, “FT3”, “FT4”, and “thyroid cancer/carcinoma” (latest search was updated on March 31st, 2022). This study also used a reference list of published papers and literature reviews to complement other relevant studies. Relevant articles were evaluated by checking their titles and abstracts, and all of the articles that met the “inclusion criteria” were included in this study.

### Inclusion criteria

The studies that were included in this meta-analysis met all of the following criteria: (1) epidemiological studies (cross-sectional study, case–control study, or cohort study) on preoperative serum thyroid-related hormone concentrations and TC risk in the population; (2) reports of the odds ratio (OR) or relative risk (RR) estimates with the corresponding 95% confidence intervals (CIs) or having sufficient information to calculate them; (3) having clear sample size information; and (4) original articles published in English. The following study types were excluded: systematic reviews; meta-analyses, case reports, and case series. Eligible studies were screened by two authors. If there was a disagreement, it was assessed and resolved by a third person.

### Data extraction

Two authors carefully extracted data from all of the eligible studies according to the inclusion criteria. If two authors could not reach a consensus, a third author was added to evaluate the article. The following data were extracted from each study: first author name, year of publication, country, cancer type, quality score, number of cases and total population, thyroid-related hormone levels, and model correction factors, and other factors.

### Quality score assessment

Three authors independently assessed the quality of the selected studies according to the Newcastle–Ottawa Scale (NOS) (16), which is frequently used for observational studies. The NOS has a total of 9 questions that assess three aspects of study subjects, the comparability between groups, and the measures of exposure. Total quality scores ranged from 0 to 9, and a higher score indicated a better quality. Disagreements were settled *via* discussion.

### Statistical methods

The meta-analysis was performed with the use of STATA 12.0 (StataCorp LP, College Station, Texas) and R software to facilitate the pooling of results across the studies.

The strength of the association between thyroid-related hormones and the risk of TC was assessed by using odds ratios with 95% confidence intervals. In the overall meta-analysis, A Z-test was used to determine the statistical significance of summary ORs, and *P*<0.05 was considered to be statistically significant. The heterogeneity among the different studies was verified by using the chi-square-based Q-test. If *P*>0.10, which indicated a lack of heterogeneity, the fixed-effect model was used. Otherwise, the random-effect model (the DerSimonian and Laird method) (17)could be used. A meta-regression analysis was performed to explore the sources of heterogeneity in the studies. The source of the control is important to the results of the study. In addition, there are differences in the distribution of TC between countries, and the incidence of TC is increasing rapidly in China (18). Thus, subgroup analyses were performed by using the control source (thyroid benign control/healthy control) and study population(Chinese/non-Chinese). Dose–response meta-analysis was performed by performing trend estimate on pooled dose–response data *via* the GLST command and generalized least squares, based on the Greenland and Rannecker method (19). Restricted cubic splines were used to evaluate the potential curvilinear relations, with knots as a nonlinear spline inflection point to model the change in dose–response risk at different serum thyroid-related hormone concentrations.

The publication bias was estimated *via* a funnel plot and Egger’s linear regression test. *P* <0.05 in the Egger’s linear regression was considered to indicate the presence of a potential publication bias. In the sensitivity analysis, we deleted one single study each time, and the new result reflected the influence of the individual dataset on the pooled OR.

## Results

### Literature search and study characteristics

**Figure 1:**
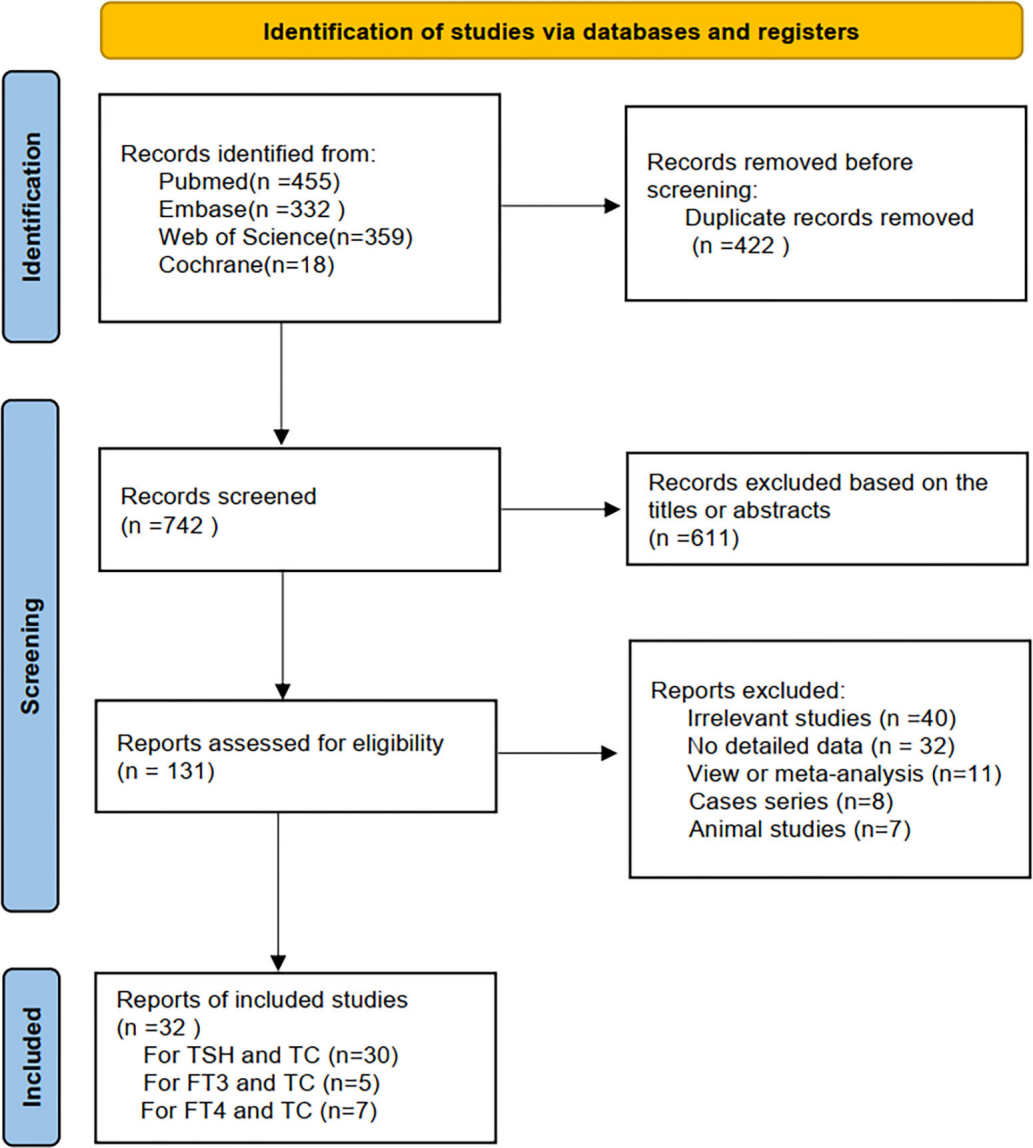
Flow diagram of study selection process.

By using different keyword combinations, 1164 articles were searched from database (PubMed: 455, Embase:332, Web of science:359, and Cochrane:18). After duplicate studies were removed (n =422), 742 remaining studies were screened. 611 articles were excluded after screening the titles and abstracts. After further review of the full text, 98 articles were excluded (40 studies were irrelevant, 32 studies were not had detailed data, 11 studies were review or meta-analyses, 8 studies were cases reports and 7 studies were animal studies). Finally, 32 articles met the inclusion criteria. 30, 5, and 7 articles were included in the meta-analysis of TSH, FT3, FT4, and TC risk, respectively. The process of literature screening is shown in **Figure 1**. The main characteristics of the studies are shown in **Table 1**. The NOS was used to assess the quality of the included studies. All of the NOS scores of the eligible studies ranged from 5 to 9, with an average of 7.5. This indicated a good quality for the meta-analysis.

**Table 1:** Overview of included studies.

Author(year)	Country	Study design	Cases/total (n)	Control group	Casetype	Thyroid-related hormones concentrations* (case/control)	Covariates adjusted in models	Quality score
Polyzos (2008) (20)	Greece	Cs	36/383	TBP	PTC, FTC	TSH : NA	Gender, solitary nodule, age.	8
Jonklaas (2008) (21)	USA	Cs	17/50	TBP	TC	TSH:1.50 ± 0.59/1.01 ± 0.51 FT4:1.04 ± 0.17/1.06 ± 0.19	NA	7
Haymart (2008) (22)	UK	Cs	212/735	TBP	DTC	TSH:2.5 ± 0.3/1.6 ± 0.1	gender, age, nodule size	9
Fiore(2009) (23)	Italy	Cs	504/10178	TBP	PTC	TSH:1.10(0.70-1.70)/0.70(0.30-1.20) FT4:10.9(9.7-12.5)/10.9(9.6-12.4) FT3:3.6(3.1-3.9)/3.6(3.1-4.0)	NA	7
Gul (2010) (24)	Turkey	Cs	166/441	TBP	TC	TSH:1.66 ± 0.96/1.09 ± 0.73 FT3:3.32 ± 0.60/3.53 ± 0.68 FT4:1.29 ± 0.24/1.34 ± 0.21	Sex, age, nodule type	8
Fiore(2010) (25)	Italy	Cs	1275/27914	TBP	PTC	TSH : NA	NA	8
Kim(2010) (26)	Korea	Cs	296/1638	TBP	TC	TSH:2.5 ± 2.8/2.1 ± 2.0	Age sex size, non-solitary nodule, thyroglobulin antibody postive	8
Dorange(2011) (27)	France	Cs	47/94	TBP	DTC	TSH:1.55 ± 0.90/0.96 ± 0.52	nodule size, ultrasound features suggestive of malignancy	9
Shi (2012) (28)	CHINA	Cs	269/1870	TBP	DTC	TSH:1.57(0.91-2.39)/1.08(0.62-1.77)	Gender, age, solitary nodule, Microcalcification, Hypoechogenicity	5
Zafon(2012) (29)	Spain	Cs	76/386	TBP	PTC,FTC	TSH:1.8 ± 0.8/1.47 ± 0.8	NA	6
Moon (2012) (30)	Korea	Cs	42/483	TBP	TC	TSH: 2.20 ± 0.15/1.87 ± 0.05 FT4: 1.26 ± 0.04/1.25 ± 0.01	Gender, age, solitary nodule, size of nodule	9
Lee (2012) (31)	Korea	Cs	35/164	TBP	PTC,FTC	TSH:1.2 ± 1.1(MIFTC);1.5 ± 0.7(WIFTC);2.4 ± 1.5(Follicular variant PTC); 1.6 ± 1.3(PTC)/1.6 ± 1.2	Age, gender, nodule size, hashimoto’s thyroiditis, irregular margin, calcification, hypoechogenicity, hypoechoic rim	7
Kim (2013) (32)	Korea	Cs	1759/3307	HC	DTC	TSH:1.95 ± 0.9/1.62 ± 0.8	age, sex, and the family history of thyroid cancer	9
Lun (2013) (33)	China	Cc	676/2478	TBP	PTC	TSH:2.02 ± 1.76/1.46 ± 1.21 FT3:4.28 ± 0.62/4.37 ± 0.61 ^a^ FT4:13.71 ± 2.09/13.74 ± 2.01 ^a^	HT, sex, age, TGAB, and TPOAB	9
Rinaldi (2014) (8)	France	Ncc	356/1120	HC	Non-rare TC	TSH: 1.15(0.67-1.83)/1.30(0.78-2.00)(W) 0.85(0.56-1.39)/1.19(0.81-1.64)(M) FT3: 3.95(3.55-4.47)/3.93(3.43-4.43)(W) 4.79(4.29-5.13)/4.72(4.32-5.11)(M) FT4: 14.82(13.46-16.26)/14.89(13.61-16.47) (W) 15.94(14.07-17.74)/15.52(14.33-17.19) (M)	on EPIC recruitment center; sex; age; date and time of the day at blood donation; fasting status;women menopausal status, sex hormone use, and phase of menstrual cycle among premenopausal women, height and weight	8
Wu (2014) (34)	China	Cs	537/2132	TBP	PTC	TSH:1.83 ± 0.07/1.39 ± 0.03 FT3:4.38 ± 0.04/4.48 ± 0.02 ^a^ FT4:13.92 ± 0.11/13.96 ± 0.03 ^a^	Age, gender, TgAb ± TN, TPOAb± TN, Tab ± TN	7
Cho (2014) (35)	Korea	Ncc	257/514	HC	TC	TSH: <1.36: 84(33.1)/59(23.2) 1.36-<2.5: 86(33.9)/111(43.7) ≥2.5: 84(33.1)/84(33.1) FT4: <1.25: 85(33.1)/68(26.5) 1.25-<1.39: 85(33.1)/75(29.2) ≥1.39: 87(33.9)/114(44.4)	age, sex, BMI, and smoking	9
Sohn (2014) (36)	Korea	Cs	2881/ 3791	TBP	PTC	TSH: 2.31 ± 0.05/1.66 ± 0.05	Gender, increasing age, solitary nodule, presence of hashimoto’s thyroiditis	7
Wang (2014) (37)	China	Cc	103/409	HC	TC	TSH:2.69 ± 0.86/2.28 ± 0.74 FT4:16.99 ± 2.72/17.1 ± 3.21 FT3: 4.08 ± 1.23/4.20 ± 1.02	TgAb, TPOAb, calcification type, patient age and sex, and urine iodine and thyroid nodule size	7
Qin (2015) (38)	China	Cs	237/1638	TBP	DTC	TSH:1.53(0.88-2.42)/1.05(0.64-1.78)	Age, sex, solitary nodule, size, TgAb ≥ 40 IU/mL, TPOAb ≥ 50 IU/mL	7
Hwang (2015) (39)	Korea	Cs	100/214	TBP	PTC	TSH:1.89 ± 1.31/5.5 ± 10.5(FLT),1.8 ± 1.3(AH) FT4:1.55 ± 1.44/1.3 ± 1.1(FLT),1.4 ± 1.3(AH)	Tg-Ab, TPO-Ab, taller than wider, absence of calcification, presence of DTD pattern	7
Hwang(2016) (40)	Korea	Cs	579/1254	TBP	TC	TSH:1.6 ± 0.9/1.7 ± 0.9	Age, gender, family history, C-statistics	7
Baser (2016) (41)	Turkey	Cs	585/1433	TBP	TC	TSH:1.46(0.40-4.04)/1.12(0.40-4.04) FT3:3.20(1.63-4.77)/(3.28(1.57-4.77) FT4:1.17(0.81-1.78)/1.16(0.85-1.78)	Nodule size, Anti- TPOAb positivity, Anti-TgAb positivity, Presence of HT	8
Huang (2017) (7)	US	Ncc	741/741	HC	TC	TSH: NA FT4: NA	BMI and branch of military service	9
Zeng (2018) (42)	China	Cs	129/258	TBP	PTC	TSH: <0.3: 6(5.3)/7(6.3) 0.3-5.5: 89(78.1)/98(87.5) >5.5: 19(16.7)/7(6.3)	NA	8
Swan (2018) (43)	Denmark	Cc	265/998	TBP	DTC	TSH: 1.3 (0.9–1.9)/0.9 (0.6–1.5)	Age, sex, Scintigraphy: cold nodule, Morphology on US	8
Zhao (2018) (44)	China	Cs	1120/2041	TBP	PTC	TSH:1.547(1.114-2.289)/1.518(0.994-2.113)(M) 1.791(1.085-2.609)/1.596(0.979-2.359)(W) FT3:4.7(4.3-5.1)/4.6(4.3-5.0)(M) 4.3(4.0-4.7)/4.4(4.1-4.8)(W) FT4: 14.1(12.9-15.4)/14.3(12.9-15.1)(M) 14.2(13.1-15.4)/14.3(13.1-15.8)(W)	age, TPOAb, TGAb, UIC, nodule size and number	8
Liu(2018) (45)	China	Cs	524/2984	TBP	TC	TSH:1.63(0.89-2.66)/1.19(0.59-2.10) FT3:4.44(3.96-5.00)/4.43(3.91-5.07) FT4:15.76(13.6-18.0)/14.97(12.84-17.42)	Age, nodule size, TGAb, TPOAb, irregular margin, solid structure, hypoechogenicity, microcalcification, macrocalcification, central flow	7
Hu (2019) (46)	China	Cs	320/329	HC	PTC	TSH:2.56(2.97)/2.42(2.05) FT3:4.56(0.70)/4.76(0.76)^a^ FT4:17.11(5.34)/16.20(2.66) ^a^	Sex, age, average annual household income, BMI, physical activity, history of CT scan, diabetes, family history of thyroid diseases, fasting serum glucose, and urinary iodine/creatinine ratio	9
Guo (2019) (47)	China	Cs	153/258	TBP	PTC	TSH:1.82(1.17-2.67)/1.40(0.78-2.09) FT3:4.86 ± 0.71/4.78 ± 0.70 FT4:15.54 ± 2.66/15.39 ± 2.05	fasting insulin, HOMA-IR, and TPOAb	7
Adhami (2020) (48)	Australia	Cs	39/104	TBP	TC	TSH:1.39 ± 1.38(T)	NA	8
Rianto (2020) (49)	Indonesia	Cs	40/80	TBP	DTC	TSH:1.24 ± 0.71/0.56 ± 0.31 FT4:0.97(0.17)/1.19(0.19)	NA	8

Cs, cross-sectional study; Cc, case-control study; Ncc, nested case-control study. W, women; M, men; T, total subjects; TBP, Thyroid Benign patients; HC, healthy controls.

*: Thyroid-related hormone concentrations presented as: Median (P25-P75)/ $\bar{x}$ ±s/mean(SD)/mean/n(%);

Unit of TSH: lIU/mL; Unit of FT3: pg/mL; Unit of FT4: ng/dL; a: unit was pmol/L.

### TSH and TC risk

A total of 30 studies with 14,487 cases and 43,950 controls were included in the meta-analysis for serum TSH and TC risks. Most of the studies were retrospective cross-sectional studies. The controls in 25 studies were benign thyroid patients, and 5 studies included healthy controls. Moreover, 10 studies were conducted in the Chinese population. Serum TSH was a continuous variable in 12 studies, a binary variable in 4 studies, and a multiclass variable in 14 studies.

**Figure 2:**
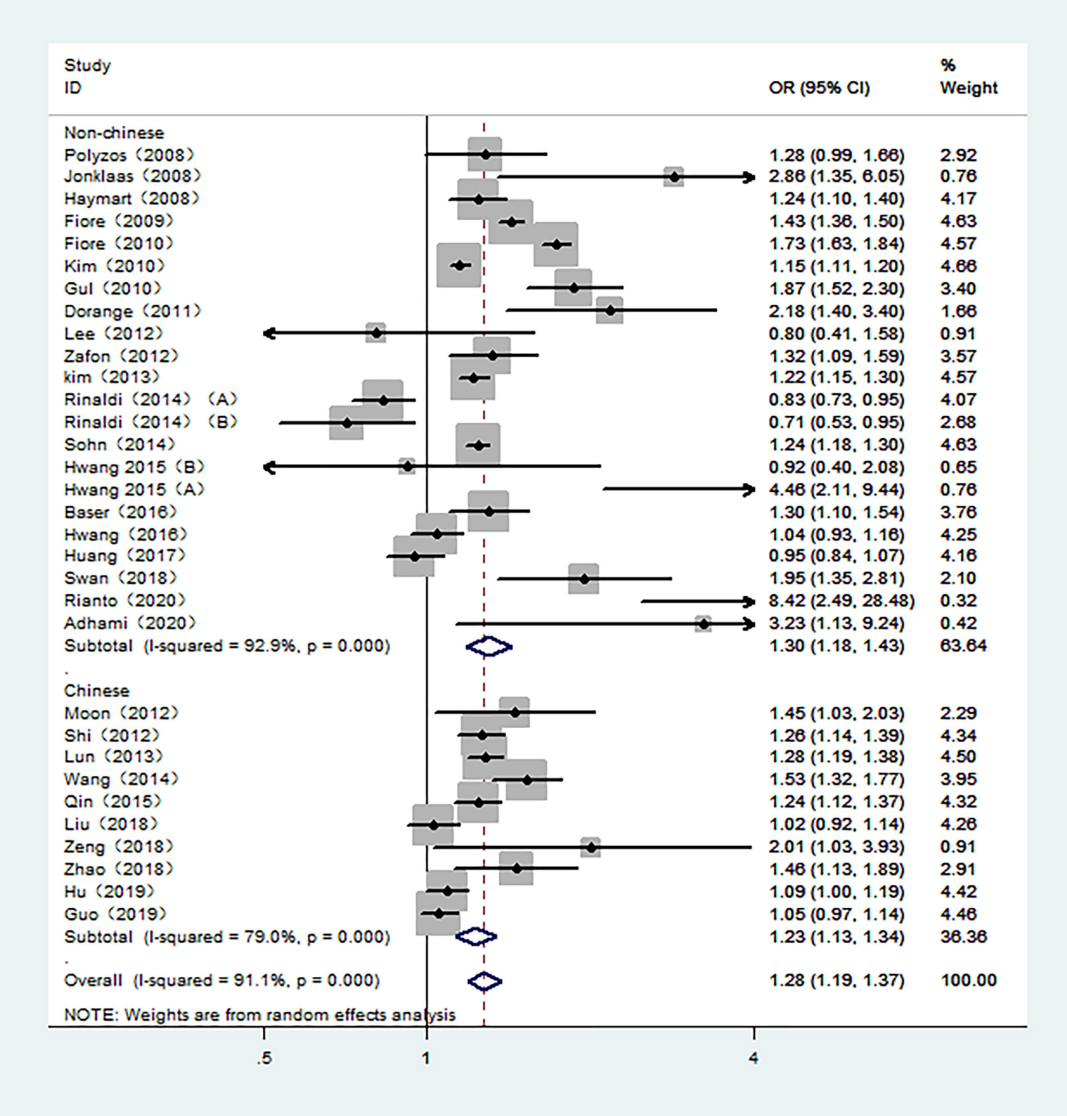
Forest plot of the risk of TC associated with TSH. Hollow diamonds represent pooled OR. Error bars indicate 95% CI.

The overall meta-analysis demonstrated an increased risk of TC associated with high TSH exposure based on the random-effect model (pool OR=1.28, 95% CI: 1.19-1.37, I^2 =^ 91.1%). Similar results were also found in studies conducted in Non-Chinese and Chinese population in random-effects models (pool OR=1.30, 95% CI:1.18-1.43, I^2 =^ 92.9%; pool OR=1.23, 95% CI:1.13-1.34, I^2 =^ 79.0%, respectively) (**Figure 2**). However, the subgroup analysis of the control sources showed that the association between TSH level and the risk of TC was only statistically significant when benign thyroid patients were used as the control group (pool OR=1.35, 95% CI: 1.25-1.46, I^2 =^ 90.2%). For those studies with healthy individuals as controls, there was no statistical relationship between the risk of TC and TSH levels (pool OR=1.04, 95% CI: 0.89-1.23, I^2 =^ 92.0%) (**Figure S1**).

14 studies with TSH as a multiclass variable were further examined for the dose–response relationship of TC risk and TSH. Subsequently, a nonlinear relationship was found. Within a given range of serum TSH concentrations, the risk of TC increased as TSH concentrations increased. According to the fractional polynomial model, the OR estimates for TC were 2.08 (95% CI: 1.48-2.91) and 2.71 (1.87-3.89) when serum TSH levels were 2.0 mU/L and 4.0 mU/L, respectively. In addition, this study also conducted a meta-analysis of 12 studies in which TSH was a continuous variable. The results showed that for every 1 mU/L increase in TSH, the risk of TC increased by 16% (pooled OR=1.16, 95% CI: 1.03-1.29).

### FT3, FT4, and TC risk

**Figure 3:**
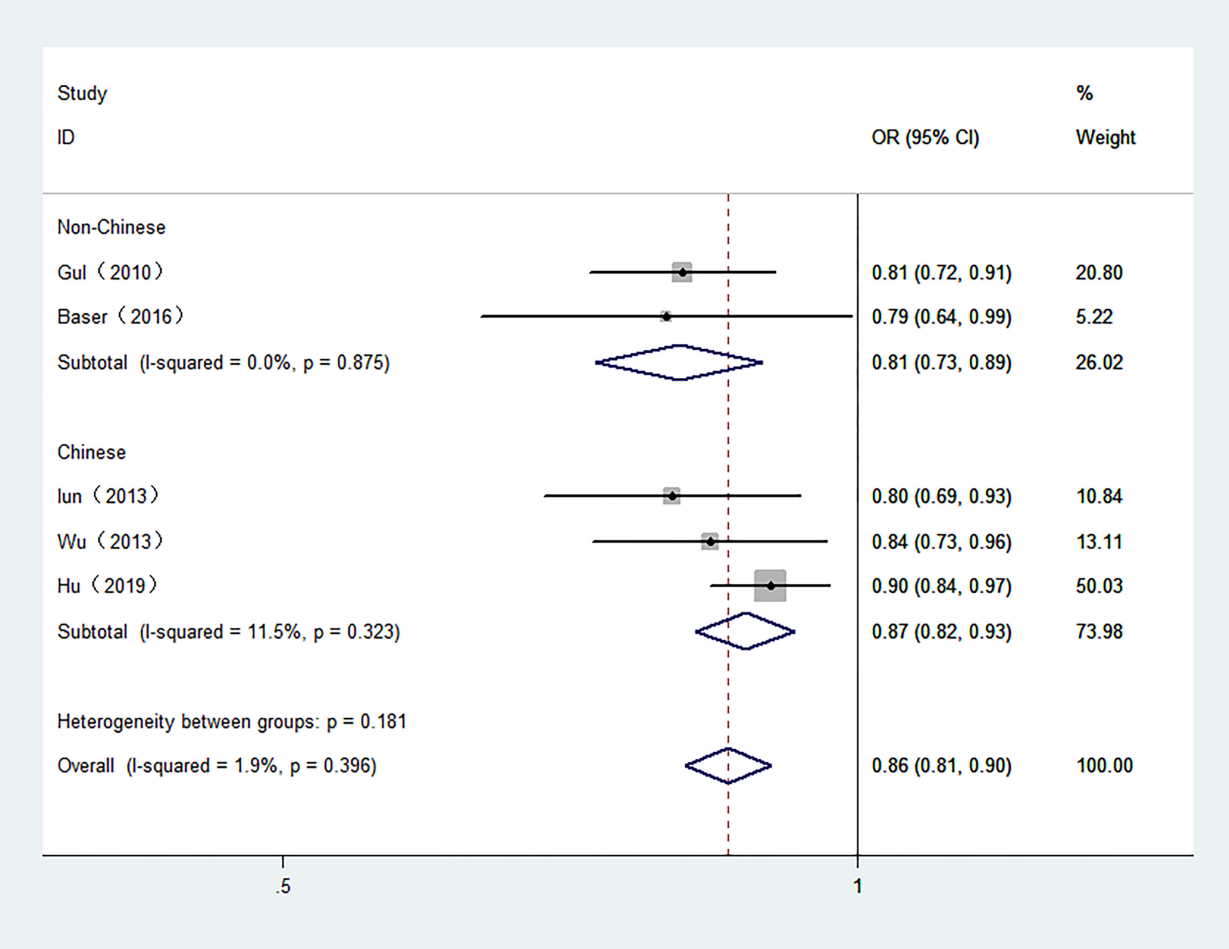
Forest plot of the risk of TC associated with FT3. Hollow diamonds represent pooled OR. Error bars indicate 95% CI.

5 studies involving 6,813 participants were included in the meta-analysis of serum FT3 and TC. The control groups in 4 studies included benign patients. Moreover, 3 studies were conducted in Chinese population. As shown in **Figure 3**, high serum FT3 demonstrated a reduced risk of TC. The combined OR was 0.86 in the fixed-effect model (95% CI: 0.81–0.90, I2 = 1.9%) in overall meta-analysis.

**Figure 4:**
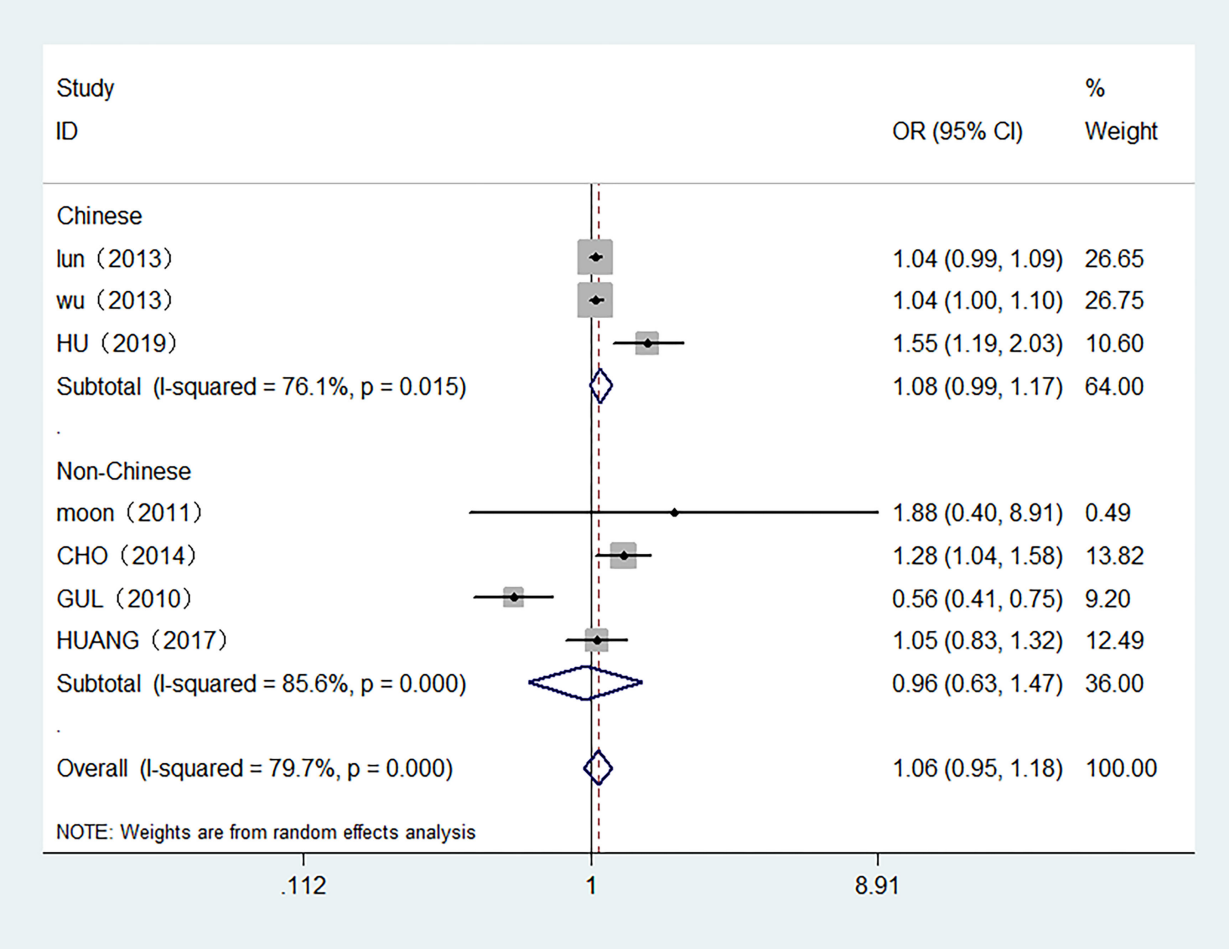
Forest plot of the risk of TC associated with FT4. Hollow diamonds represent pooled OR. Error bars indicate 95% CI.

**Figure 5:**
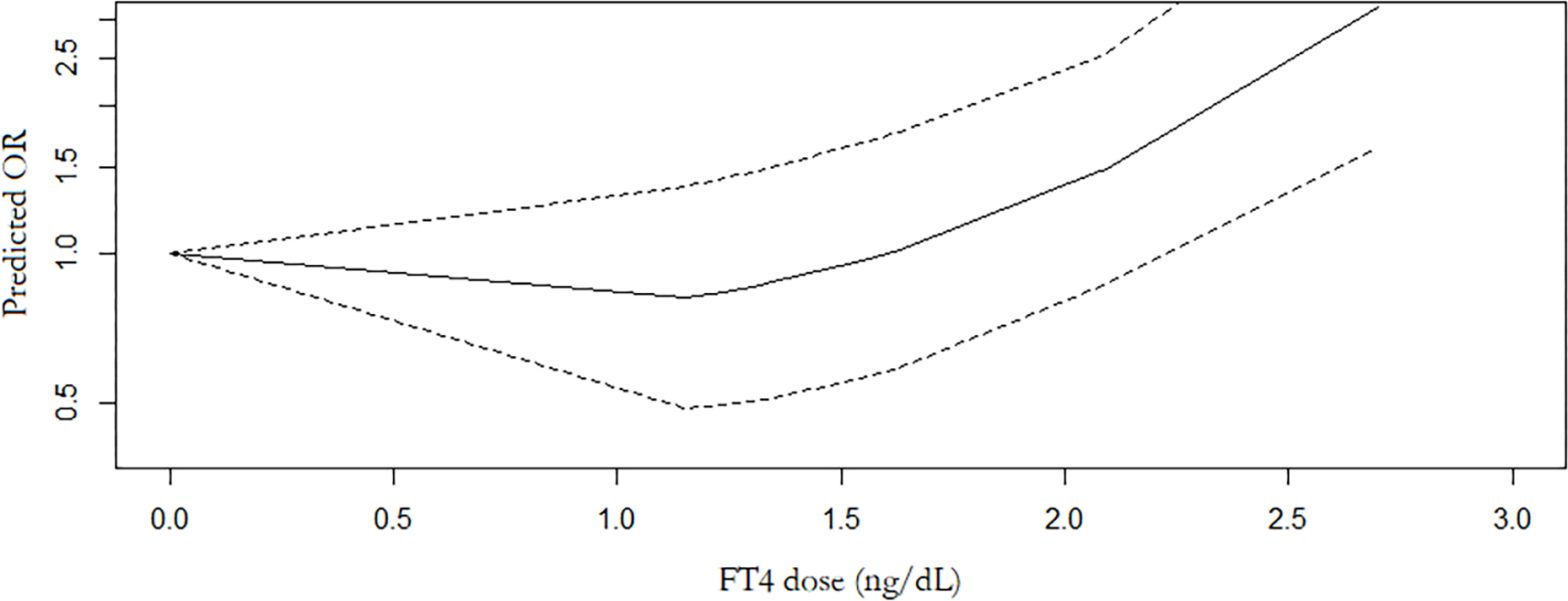
Dose response relationship between FT4 level and TC risk.

7 studies with 7,118 participants were included in the meta-analysis of serum FT4 and TC. 3 studies were conducted in Chinese population. The overall meta-analysis results are shown in **Figure 4**. We failed to find any relationships between the FT4 level and the risk of TC in the random effect model. Similar results were also shown in the stratified analysis. The OR for TC in relation to FT4 was not significant in either the Chinese or Non-Chinese population groups. 4 studies with FT4 as a multiclass variable were further examined for the dose–response relationship of the risk of TC and FT4, and a nonlinear relationship was found (P <0.001). **Figure 5** displays the predicted OR and 95% CI of TC corresponding to the FT4 dose data. When the FT4 concentration reached a certain threshold (approximately 2.2 ng/dL), the risk of TC increased with statistical significance. According to the fractional polynomial model, the OR estimates for TC were 2.15 (95% CI: 1.20-3.86) and 3.32 (95% CI: 1.63-6.82) when serum TSH levels were 2.5 mU/L and 3.0 mU/L, respectively.

### Publication bias and sensitivity analysis

The Egger’s test was used to determine whether there was a publication bias in this study. The Egger’s test results were not significant for 3 overall meta-analyses (*P*=0.54 for TSH and TC; *P*=0.07 for FT3 and TC; *P*=0.82 for FT4 and TC), thus indicating that no significant bias existed. We deleted a single study involved in this meta-analysis each time to investigate the influence of single study data on the pooled ORs. The results showed that the pooled ORs did not change dominantly in any model, which indicated that the pooled estimates were stable (**Figures S2, S3**).

### Meta-regression analyses

Meta-regression analyses for the evaluation of heterogeneity were conducted to investigate whether the association between TSH, FT4, and TC risk was modified by the control source and study population. For TSH and the risk of TC, it was found that the control source explained 21.15% of the heterogeneity, whereas the study population explained 7.87% of the heterogeneity. For FT4 and the risk of TC, the study population and control source explained 27.43% and 11.4% of the heterogeneity, respectively.

## Discussion

To our knowledge, this is the first study to combine overall and dose–response analyses to comprehensively explore the association between preoperative thyroid-related hormone levels and TC risk. The results indicated that high TSH levels were related to an increased risk of TC, especially in individuals with benign thyroid disease. In addition, the risk of TC was inversely associated with the serum FT3 level. At present, the incidence of TC is rapidly increasing worldwide. This study provides a new view of the potential relationships between thyroid-related hormones and the risk of TC, which may play an important role in the prevention of TC.

TSH is a two-subunit glycoprotein produced by the anterior pituitary gland. Its main role is to regulate thyroid hormones, promote thyroid cell growth, stimulate sodium iodide transporters, and regulate extrathyroidal effects. In 2011, Moon et al (30)conducted a cross-sectional study from the population perspective for the first time and found that thyroid disease patients with higher TSH levels had a significantly higher malignancy rate of TC. Subsequent studies demonstrated the same results (28, 33, 38, 41, 48). Several meta-analyses have further reported that with increasing TSH levels, the risk of TC also increases (11, 12). However, a genome-wide association study of 22 single-nucleotide polymorphisms using the general population as controls found that common variants with low TSH concentrations were positively associated with TC (50). In previous meta-analyses, the control groups were dominated by patients with benign thyroid disease, not the general population or healthy controls (11–13). This may be part of the reason for the inconsistent conclusions. Overall, this study found that TSH levels were positively associated with the risk of TC. To understand whether different control populations had an effect on this association, a control source subgroup analysis was performed. The results showed that the association between TSH levels and the risk of TC was not statistically significant when healthy people were used as controls. In the control group with benign thyroid disease, some individuals may have thyroid nodules. The relationship between thyroid nodules and TSH is not clear. It is thought that some nodules can produce high levels of thyroid hormones, thus lowering TSH levels (7). Therefore, the TSH levels of different control sources may be different, which may lead to inconsistencies in the subgroup analysis (to a certain extent). TSH has been reported to act like a growth factor in thyroid tumors, and have a mitogenic role in the proliferation of TC cells (41, 51). DTC cells hide TSH receptors in cell membranes and respond to TSH stimulation by increasing the expression of several thyroid specific proteins and causing an increase in cell growth rate (52). Animal experiments have shown that excessive TSH can induce hyperplasia and eventually cancer (33, 53). Besides, almost all papillary and follicular carcinomas express varying levels of TSH receptor mRNA (54, 55). Therefore, TSH may play a certain role in the occurrence and development of thyroid tumours. Although this study failed to find any statistically significant associations between TSH levels and the risk of TC with healthy people as a control group, due to the small sample size and the non-prospective study design, further large-sample prospective studies are needed to verify the accuracy of this result in the future.

It has been reported that individuals with thyroid dysfunction have a higher risk of developing TC (56). However, thyroid hormone levels and TC risk have not been sufficiently studied. Studies have found that relatively high FT4 levels are positively associated with TC risk in people with normal thyroid hormone levels (56). Interestingly, Gul et al. evaluated the association between thyroid functions (FT3 and FT4) and DTC and found that lower FT3 andFT4 concentrations within normal limits were related to increased TC, independent of sex and nodule type (24). This meta-analysis found that there was a negative association between FT3 and the risk of TC. Although there was no association between FT4 and the risk of TC in the overall meta-analysis, the dose–response meta-analysis demonstrated a nonlinear relationship between FT4 and the risk of TC; when the FT4 level was higher than approximately 2.2 ng/dL, the risk of TC was significantly increased. This result was consistent with the results reported by Hu et al. (46). One of the possible mechanisms may involve the upregulation of TSH by abnormal thyroid hormone levels. In addition, thyroid hormones were also reported to be associated with cellular transformation, tumorigenesis, and metastasis (57). The T3 receptor can act on its promoter to control the expression of thyroid fibroblast growth factor. Animal experiments have indicated that mutations in the T3 receptor may increase phosphatidylinositol 3-kinase signalling and induce thyroid tumours (58). High FT4 and low FT3 levels were associated with a higher risk of TC, possibly due to disturbed expression of type 1 iodothyronine deiodinase in the development of thyroid malignancies, thus reducing the transition rate from an inactive FT4 state to an active FT3 state (59). Due to the fact that FT4 tends to be higher and FT3 tends to be lower in the case of malignant tumours, it has been suggested that the quotient (FT4/FT3) can be used to assess whether these trends increase the risk of TC (60).

This meta-analysis had several strengths. First, we used two different methods (overall and dose response meta-analyses) to investigate the association between preoperative thyroid-related hormone levels and the risk of TC. Second, compared with other meta-analyses of TSH and the risk of TC, this study was more comprehensive and provides updates for the latest literature in recent years. Additionally, the effect of thyroid hormones on the risk of TC was analysed for the first time. Our meta-analysis also had some limitations. For example, nonpublished data, non-English-language studies, and missed studies may exist and may have influenced our results. Besides, retrospective studies cannot determine the causal link between thyroid-related hormones and disease risk. Furthermore, the control population included in the study may differ in terms of country, health status and demographic characteristics, which may confound the results. Subgroup analysis was conducted for the source of controls in this study but does not fully explain the heterogeneity of the findings. Last but not the least, TC predominates in women and has been trending younger in recent years. Due to limited relevant studies, subgroup analysis by gender and age was not performed in this study. Whether there are gender, age, and other demographic differences in the association between thyroid-related hormones and TC risk needs to be further explored.

## Conclusions

In conclusion, the existing data included in this meta-analysis provide evidence favouring a significant relationship between high TSH and FT4 concentrations, and low FT3 concentrations contribute to the increased risk of TC. Further prospective research is required to clarify the impact of thyroid-related hormones on the initiation of TC.

## Data availability statement

The original contributions presented in the study are included in the article/**Supplementary Material**. Further inquiries can be directed to the corresponding authors.

## Author contributions

YW collected data and drafted manuscripts. YL and ZW completed the search and selection of the database, reviewed the articles and extracted the data. QZ analysed the data and submitted the paper. RF and YJ designed the study and revised the article. All authors contributed to the article and approved the submitted version.
