# Supplementary material for: The associations between thyroid-related hormones and the risk of thyroid cancer: An overall and dose-response meta-analysis
Source: Front Endocrinol (Lausanne). 2022 Dec 7;13:992566. doi: 10.3389/fendo.2022.992566 (PMC9768331; doi:10.3389/fendo.2022.992566)
Supplement: Supplementary file 1 [file DataSheet_1.docx]

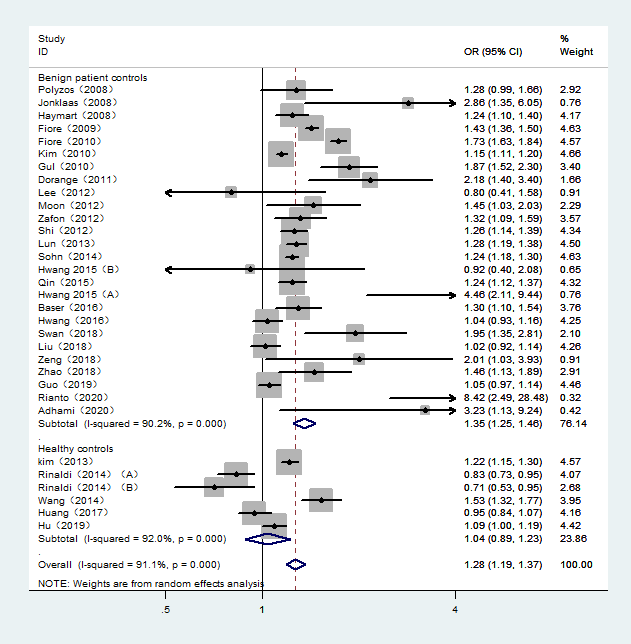


**Figure S1:** Forest plot of the risk of TC associated with TSH in different control sources. Hollow diamonds represent pooled OR. Error bars indicate 95% CI.


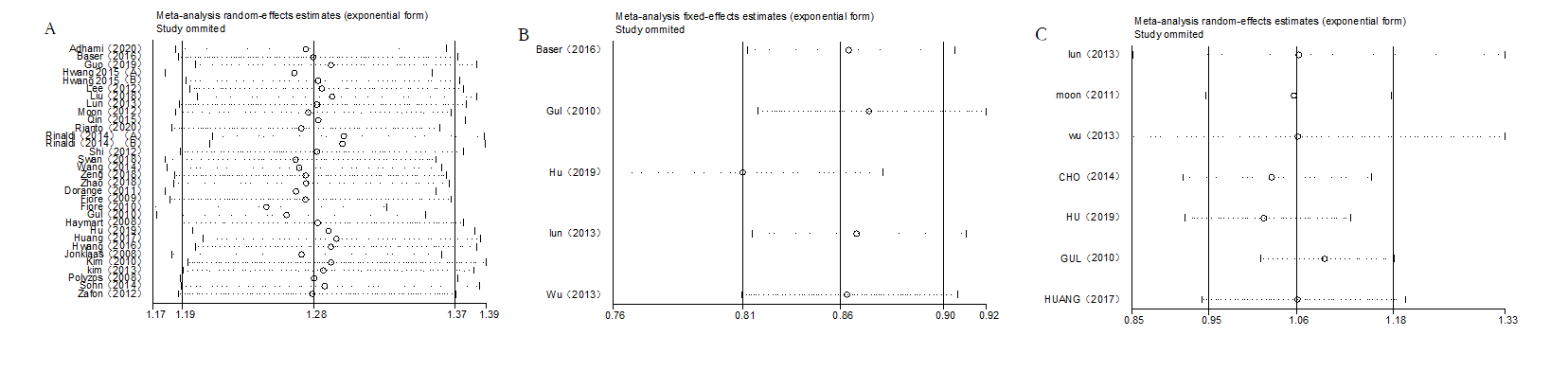


**Figure S2** Sensitivity analysis plot: investigated the influence of a single record on the overall OR estimate by removing each record in each turn. A: TSH and TC risk; B: FT3 and TC risk; C:FT4 and TC risk.


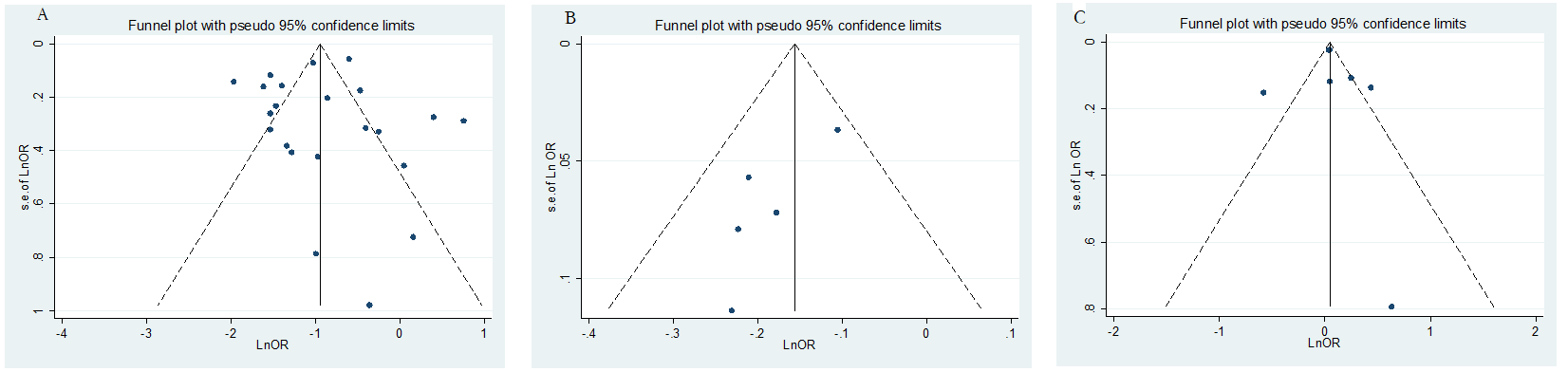


**Figure S3** Funnel diagram of meta analysis; A: TSH and TC risk; B: FT3 and TC risk; C:FT4 and TC risk.
